# Changes in Intra-Amniotic, Fetal Intrathoracic, and Intraperitoneal Pressures with Uterine Contraction: A Report of Three Cases

**DOI:** 10.1155/2018/4281528

**Published:** 2018-09-12

**Authors:** Daisuke Katsura, Yuichiro Takahashi, Shigenori Iwagaki, Rika Chiaki, Kazuhiko Asai, Masako Koike, Shunsuke Yasumi, Madoka Furuhashi

**Affiliations:** Department of Fetal-Maternal Medicine, Nagara Medical Centre, 1300-7, Nagara, Gifu 502-8558, Japan

## Abstract

Intra-amniotic, fetal intrathoracic, and intraperitoneal pressures during pregnancy have been previously investigated. However, to our knowledge, changes in these pressures during uterine contractions have not been reported. Herein, we present three cases of polyhydramnios, fetal pleural effusion, and fetal ascites, in which intra-amniotic, fetal intrathoracic, intraperitoneal pressures increased with uterine contractions. These pressure increases may affect the fetal circulation. We suggest that managing potential premature delivery (e.g., with tocolysis) is important in cases with polyhydramnios and excess fluid in fetal body areas, such as the thorax, abdomen, and heart. The results of this preliminary study on intrafetal pressure measurements will be useful in performing fetal and neonatal surgeries in the future.

## 1. Introduction

Caldeyro-Barcia and Poseiro (1960) reported that uterine contractions (UCs) increases human intrauterine pressure [1, 2]. Intra-amniotic pressure during pregnancy has been reportedly measured via a transabdominal approach [3, 4]. However, to our knowledge, direct measurement of pathologic fetal intrathoracic and intraperitoneal pressures during pregnancy has not been systematically reported [5, 6]. In addition, association between fetal pressure and UCs has not been reported.

UCs cause a significant reduction in placental perfusion [7], and the association between impaired placental perfusion and fetal growth restriction is known [8]. Several physiological studies using fetal pulse Doppler have been reported [9, 10]. Furthermore, in fetal growth restriction, the umbilical artery (UA) pulsatility index (PI) was significantly high during UCs in cases with positive oxytocin challenge test [9]. Moreover, the inferior vena cava preload index (PLI) increased with UCs in normal pregnancy [10]. Therefore, UCs may indirectly affect fetal circulation, including preload and afterload. However, the direct effect of pressures on the fetus remains unclear. This study reports three cases in which UCs increased intra-amniotic, fetal intrathoracic, and intraperitoneal pressures and discusses the potential effect of these pressures on the fetus.

## 2. Case Presentations

### 2.1. Ethical Approval

The study was accepted and approved by the Institutional Review Board and Ethical Committee of Nagara Medical Centre (IRB number: 28-19). All patients provided informed consent.

### 2.2. Case 1

**Figure 1:**
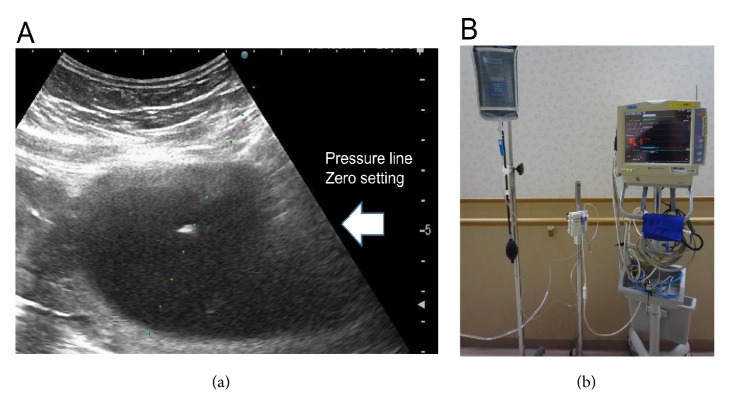
(A) Ultrasound and zero pressure level line setting during amnioreduction for polyhydramnios. (B) Circuit that includes a silicone stain-gauge transducer (DX-300, Nihon Koden) for measuring pressure.

Case 1 was a 30-year-old, gravida 2, para 1 woman. At week 22 of pregnancy, she was admitted to our hospital because of a monochorionic twin pregnancy with cervical dilatation, frequent UCs on cardiotocogram, and polyhydramnios. As intervention, tocolysis was performed. Although UCs were reduced, ultrasonography revealed a maximum vertical pocket (MVP) of 12 cm (although the MVP of the co-twin was 4 cm) and the patient had dyspnea; therefore, emergency amnioreduction was performed, and 2,000 mL of amniotic fluid was drained during tocolysis. Intra-amniotic pressure was measured during the procedure. A saline-filled line was attached at one end to the hub of the needle and at the other end to a silicone stain-gauge transducer (DX-300; Nihon Kohden Corporation, Tokyo, Japan). Readings were recorded at the needle tip and were recorded if they were stable for 10 s. A zero pressure level line setting was performed at the estimated vertical line using the ultrasound-guided needle tip level (Figure 1). Prior to this case, we did not perform this procedure for pressure measurement alone.

The intra-amniotic pressure was 16 mmHg before reduction, increased to 29 mmHg with UCs during reduction, and then declined to 9 mmHg after reduction. At this point, the Doppler of the recipient and donor showed the following results: UA PI, 1.42 and 1.54; middle cerebral artery (MCA) PI, 1.56 and 2.79; umbilical venous flow volume (UVFV), 149 and 110 mL/kg/min; ductus venosus (DV) PI, 0.74 and 0.65; cardiothoracic area ratio (CTAR), 24 and 30%; inferior vena cava PLI, 0.26 and 0.34. These data were within the normal limit, but the recipient had moderate tricuspid regurgitation. After amnioreduction, the Doppler of the recipient and donor showed the following results: UA PI, 1.18 and 1.79; MCA PI, 1.59 and 2.79; UVFV, 225 and 114 mL/kg/min; DV PI, 0.81 and 0.70. These data were within the normal limit, but the recipient's UA PI decreased mildly and UVFV increased. At week 25 of pregnancy, the MVPs of the donor and recipient were 1 and 15 cm, respectively. Therefore, the patient was diagnosed with stage II twin-to-twin transfusion syndrome, and fetoscopic laser photocoagulation was performed. Thereafter, caesarean section was performed due to labor onset at week 28 of pregnancy. Male neonates were born weighing 1573 and 1709 g, with Apgar scores of 5 and 4 at 1 min and 6 and 4 at 5 min, and umbilical arterial cord blood pH of 7.312 and 7.264, respectively. They were admitted to the neonatal intensive care unit because of prematurity and low birth weight and respiratory distress syndrome. The neonates responded well to the treatment.

### 2.3. Case 2

Case 2 was a 29-year-old, gravida 1, para 0 woman. Fetal ascites was observed at week 33 of pregnancy, and the patient was admitted to our hospital. Test results for maternal serum cytomegalovirus and parvovirus B19 IgM were negative. Abdominocentesis was performed for diagnosis, and 99 mL of ascitic fluid was drained, containing 92% lymphocytes; hence, diagnosis of chylous ascites was made. The subsequent pregnancy course was uneventful until term. However, abdominocentesis was performed again to reduce the risk of dystocia [11] at week 37 of pregnancy, with drainage of 510 mL of ascitic fluid during labor preparation. Tocolysis was performed for preventing UC only during the procedure. The fetal intraperitoneal pressure was 18 mmHg before drainage, 14 mmHg after drainage, and 32 mmHg when confirming UC on palpation. At this point, the fetal Doppler showed the following results: UA PI, 0.98; MCA PI, 1.08; and DV PI, 0.87. These values were within the normal limit. After abdominocentesis, the fetal Doppler showed the following results: UA PI, 0.65; MCA PI, 1.04; and DV PI, 0.59. These data were within the normal limit, but UA PI decreased. Thereafter, ascites recurred, and the mother had spontaneous premature rupture of membranes at week 38 of pregnancy. Caesarean section was performed due to cephalopelvic disproportion. A male neonate was born weighing 3117 g, with Apgar scores of 8 at 1 min and 9 at 5 min and umbilical arterial cord blood pH of 7.221. He was admitted to the neonatal intensive care unit for chylous ascites. Ascitic fluids were drained twice. However, although there was ascitic fluid retention, the amount did not increase. He was transferred to another hospital.

### 2.4. Case 3

Case 3 was a 30-year-old, gravida 1, para 1 woman. Fetal pleural effusion was observed at week 22 of pregnancy during a routine prenatal visit, and she was admitted to our hospital. Skin edema and ascites were not observed. Thoracentesis was performed, and 24 mL of the pleural fluid was drained, containing 95% lymphocytes; hence, a diagnosis of chylothorax was made. Although the amniotic fluid index was normal, the intra-amniotic pressure remained as high as 20–22 mmHg because of frequent UCs on palpation even if tocolysis was performed for preventing UC during the procedure. The fetal intrathoracic pressure was 30 mmHg before and 19 mmHg after drainage. At this point, the fetal Doppler showed the following results: UA PI, 1.39; MCA PI, 1.57; UVFV, 72 mL/kg/min; DV PI, 0.58; CTAR, 17.1%; inferior vena cava PLI, 0.4; and TEI index (left/right ventricle), 0.474/0.581.These values indicate that CTAR was low and the right ventricle TEI index was mildly high. After thoracentesis, the fetal Doppler showed the following results: UA PI, 1.26; MCA PI, 1.42; UVFV, 73 mL/kg/min; DV PI, 0.63. These data were within the normal limit and did not change. Because pleural effusion recurred within 7 days after the first procedure, a thoracoamniotic shunt was inserted into the left pleural space 4 days after the first procedure according to the Japanese protocol for thoracoamniotic shunt [12]. Thereafter, the pleural effusion reduced and did not recur. Consequently, she was transferred to another hospital at week 26 of pregnancy.

## 3. Discussion

Few reports have assessed intra-amniotic, fetal intrathoracic, and intraperitoneal pressures.  Table 1 presents a review of the previously reported pressures, as well as those presented in our cases [4–6]. Normal intra-amniotic pressure during pregnancy is believed to exponentially decrease with gestation, from 9 mmHg at 10 weeks to 5 mmHg at 30 weeks [3]. Additionally, normal intra-amniotic pressure was not significantly elevated in twin pregnancies [4]. Reports on fetal intrathoracic pressure in cases of congenital chylothorax revealed a correlation between increased intrathoracic pressure and ultrasonographic signs of mediastinal shift and diaphragm inversion [5]. Fetal intraperitoneal pressure during intrauterine transfusion in patients with Rhesus alloimmunization showed a basal pressure of 2.5 mmHg (95% confidence interval, 1.4–3.6), and the pressure in complicated intraperitoneal transfusion significantly increased compared to that in uncomplicated pregnancies [6]. To our knowledge, the association between fetal pressure and UCs has not been previously reported.

Pressures were previously measured near the uterine fundus [3, 4, 6]; however, in the present study, these pressures were measured at the tip of the needle, based on the methods used to measure adult central venous pressure [13, 14]. Considering these factors, our measured pressures were higher (approximately 3–5 mmHg) than those previously reported. Moreover, there was no difference in the normal control of intra-amniotic pressure between our methods (16 and 18 weeks; mean, 10.75 mmHg; range, 10–3 mmHg; four cases were measured during amniocentesis for amniotic diagnosis) and those of previous reports (16 and 18 weeks; lower limits, 2.2 and 2.2 mmHg, respectively; upper limits, 9.3 and 9.5 mmHg, respectively) [4], which suggests that our methodology is reliable (Table 1).

In our cases, intra-amniotic pressures increased with UCs and were higher than the normal range in cases with polyhydramnios [4]. Intra-amniotic pressure may increase because the intrauterine pressure caused by UCs exceeds the uterine tolerance and the amniotic fluid cannot escape. Intra-amniotic pressure >15 mmHg may be perceived as painful and uncomfortable if polyhydramnios is present [1, 2]. For example, in Case 1, the pressure was 16 mmHg and the mother had dyspnea. After amnioreduction, the pressure returned to the normal range, and the dyspnea resolved. According to Nicolini et al. [6], the increase in pressure due to excess fluid should return to the normal range once the excess fluid is removed. However, in Case 2, although the fluid was removed, the pressure remained higher than the normal range, possibly because the intra-amniotic pressure was increased due to polyhydramnios. Further, to our knowledge, normal fetal intrathoracic pressures have not previously been reported. In Case 3, after the pleural effusion was drained, intrathoracic pressure decreased and was equal to the intra-amniotic pressure. This may have occurred because the intra-amniotic pressure increased as a result of frequent UCs, which ultimately affected intrathoracic pressure.

Even with the amniotic fluid as cushioning, UC pressure on the fetus increased, as did the intrathoracic and intraperitoneal pressures. According to Pascal's principle, a change in pressure at any point in an enclosed fluid compartment at rest is transmitted undiminished to all points in the fluid. Thus, the increased intrathoracic and intraperitoneal pressures equally affect the intra-amniotic pressure, which is influenced by UCs and/or polyhydramnios. These pressures on the fetus squeeze the surrounding organs and blood vessels, possibly affecting fetal circulation. In these cases, although fetal flow velocity was within the normal limit, the pressures during uterine contractions might be changing. In Case 1, decreased intra-amniotic pressure decreased UA PI and increased UVFV, and, in Case 2, decreased fetal intraperitoneal pressure decreased UA PI. These suggested to improve fetal circulation.

We believe that management of potential premature delivery with tocolysis is important in cases with polyhydramnios and excess fluid in fetal body areas, such as the thorax, abdomen, and heart. The results of this preliminary study on intrafetal pressure measurements will be useful for performing fetal and neonatal surgeries in the future, such as judgment of adaptation of fetal thoracoamniotic shunt and neonatal thoracic drainage depending on fetal intrathoracic pressure, and for removal of fluid for improving fetal circulation and facilitating neonatal resuscitation depending on intra-amniotic, fetal intrathoracic, and intraperitoneal pressure. However, further data are warranted to clarify the association between the extent of pressure and the effect on the fetus and to establish the selection criteria for fetal and neonatal surgeries.

## Figures and Tables

**Table 1:** Results and literature review of intra-amniotic and fetal intrathoracic and intraperitoneal pressures.

Case	Diagnosis	GA at measurement (weeks)	Intra-amniotic pressure (mmHg)			Intrathoracic pressure (mmHg)			Intraperitoneal pressure (mmHg)		
			Before R	After R/baseline^a^	With UC	Before R	After R	With UC	Before R	After R	With UC
1	Polyhydramnios	22	16	9	29						
2	Chylous ascites polyhydramnios	38							18	14	32
3	Chylothorax	22			20–22	30	19				
Our control cases	Amniocentesis for chromosome analysis	16		10, 10							
		18		10, 13							
Yamamoto et al. 2007	Chylothorax polyhydramnios	31	21			39					
				Baseline^a^						Baseline^a^	
Nicolini et al. 1989	Intrauterine transfusion									2.5 (1.4–3.6)	
Fisk et al. 1992		16		2.2–9.3							
		18		2.2–9.5							
		20		2.3–9.6							
		22		2.3–9.7							
		24		2.3–9.7							
		26		2.3–9.8							
		28		2.4–10.1							
		30		2.5–10.6							
		32		2.8–11.5							
		34		3.2–13.1							

GA, gestational age; R, removal of amniotic fluid; UC, uterine contraction; baseline^a^, baseline pressure not related to amnioreduction.

## References

[1] Caldeyro-Barcia R., Poseiro J. J. (1960). Physiology of the uterine contraction. *Clinical Obstetrics and Gynecology*.

[2] Young R. C. (2016). Mechanotransduction mechanisms for coordinating uterine contractions in human labor. *Reproduction*.

[3] Sideris I. G., Nicolaides K. H. (1991). Amniotic fluid pressure during pregnancy. *Fetal Diagnosis and Therapy*.

[4] Fisk N. M., Ronderos‐Dumit D., Tannirandorn Y., Nicolini U., Talbert D., Rodeck C. H. (1992). Normal amniotic pressure throughout gestation. *BJOG: An International Journal of Obstetrics & Gynaecology*.

[5] Yamamoto M., Insunza A., Carrillo J., Caicedo L. A., Paiva E., Ville Y. (2007). Intrathoracic pressure in congenital chylothorax: Keystone for the rationale of thoracoamniotic shunting?. *Fetal Diagnosis and Therapy*.

[6] Nicolini U., Talbert D. G., Fisk N. M., Rodeck C. H. (1989). Pathophysiology of pressure changes during intrauterine transfusion. *American Journal of Obstetrics & Gynecology*.

[7] Sinding M., Peters D. A., Frøkjær J. B., Christiansen O. B., Uldbjerg N., Sørensen A. (2016). Reduced placental oxygenation during subclinical uterine contractions as assessed by BOLD MRI. *Placenta*.

[8] Moran M. C., Mulcahy C., Zombori G., Ryan J., Downey P., McAuliffe F. M. (2015). Placental volume, vasculature and calcification in pregnancies complicated by pre-eclampsia and intra-uterine growth restriction. *European Journal of Obstetrics & Gynecology and Reproductive Biology*.

[9] Li H., Gudmundsson S., Olofsson P. (2006). Acute centralization of blood flow in compromised human fetuses evoked by uterine contractions. *Early Human Development*.

[10] Takahashi Y., Iwagaki S., Nakagawa Y., Kawabata I., Tamaya T. (2003). Uterine contractions increase fetal heart preload. *Ultrasound in Obstetrics & Gynecology*.

[11] Haider P., Korejo R., Jafarey S. (1991). Fetal ascites as a cause of dystocia in labour.. *Journal of the Pakistan Medical Association*.

[12] Takahashi Y., Kawabata I., Sumie M. (2012). Thoracoamniotic shunting for fetal pleural effusions using a double-basket shunt. *Prenatal Diagnosis*.

[13] Wilson J. N., Grow J. B., Demong C. V., Prevedel A. E., Owens J. C., Hamilton W. K. (1964). Central venous pressure in optimal blood volume maintenance. *Survey of Anesthesiology*.

[14] Barbeito A., Mark J. B. (2006). Arterial and Central Venous Pressure Monitoring. *Anesthesiology Clinics of North America*.

